# Single Grain Boundary Modeling and Design of Microcrystalline Si Solar Cells

**DOI:** 10.3390/ma6010291

**Published:** 2013-01-21

**Authors:** Chu-Hsuan Lin, Wen-Tzu Hsu, Cheng-Hung Tai

**Affiliations:** Department of Opto-Electronic Engineering, National Dong Hwa University, Shoufeng, Hualien 97401, Taiwan; E-Mails: m9925003@ems.ndhu.edu.tw (W.-T.H.); m9724010@ems.ndhu.edu.tw (C.-H.T.)

**Keywords:** microcrystalline silicon, grain boundary, three-terminal cell

## Abstract

For photovoltaic applications, microcrystalline silicon has a lot of advantages, such as the ability to absorb the near-infrared part of the solar spectrum. However, there are many dangling bonds at the grain boundary in microcrystalline Si. These dangling bonds would lead to the recombination of photo-generated carriers and decrease the conversion efficiency. Therefore, we included the grain boundary in the numerical study in order to simulate a microcrystalline Si solar cell accurately, designing new three-terminal microcrystalline Si solar cells. The 3-μm-thick three-terminal cell achieved a conversion efficiency of 10.8%, while the efficiency of a typical two-terminal cell is 9.7%. The three-terminal structure increased the J_SC_ but decreased the V_OC_, and such phenomena are discussed. High-efficiency and low-cost Si-based thin film solar cells can now be designed based on the information provided in this paper.

## 1. Introduction

Low cost Si-based materials, including microcrystalline silicon (μc-Si) and amorphous silicon (a-Si), are promising for use in photovoltaic applications. The μc-Si solar cell has a slight light-induced degradation so that it can stably maintain efficiency compared to the a-Si solar cell [1]. Therefore, μc-Si is a key material among several common materials for thin film solar cells. Plasma enhanced chemical vapor deposition (PECVD) and very high frequency plasma-enhanced chemical vapor deposition (VHF PECVD) are usually used to prepare μc-Si thin film solar cells [2,3]. In addition, hot wire chemical vapor deposition (HWCVD) and photochemical vapor deposition (photo-CVD) can also be used to deposit μc-Si film at a low fabrication temperature [4,5,6]. The low fabrication temperature is suitable for substrates of low cost, low melting points and with a low thermal budget. Generally, the fabrication based on HWCVD can lead to a high deposition rate and high open circuit voltage (V_OC_) [7]. For the near-infrared part of the solar spectrum, the narrow band gap of the μc-Si contributes to high quantum efficiency (QE).

Although μc-Si offers a lot of advantages for photovoltaic applications, the μc-Si film has dangling bonds at the grain boundary (GB) [8]. The conversion efficiency of solar cells is limited by recombination of photo-generated carriers at dangling bonds of the grain boundary [9]. In this paper, we therefore included the grain boundary in the numerical study. In addition, we propose a new three-terminal μc-Si structure to suppress such recombination. We investigated the two-terminal and the three-terminal μc-Si solar cells with the same material parameters and device thicknesses, utilizing the simulation tool, Sentaurus TCAD. In the tool, a device was discretized into a finite number of nodes, and material parameters—such as doping concentration—were assigned to each node. All quantities for any point between these nodes could be obtained by interpolation. A fully coupled Poisson and electron/hole continuity equations were self-consistent solved. The optical model for optical generation was raytracing, where a plane wave was partitioned into one-dimensional rays of light. The refractive index, n, and extinction coefficient, k, as a function of the wavelength for different materials were included in advance. The absorption coefficient α was calculated from the formula α = 4πk/λ, where λ was the wavelength of light. The original AM 1.5G solar spectrum was taken from [10], with wavelengths ranging from 320 nm to 2280 nm and a sampling interval of 40 nm. The μc-Si was usually deposited on glass with rough (textured) transparent conducting oxide (TCO) for the superstrate cell structure. In order to consider the reflection or scattering of light [11] at air/glass, glass/TCO, TCO/μc-Si interfaces, an optical simulation tool, RSOFT was used. First, the reflection (R) of air/glass/TCO/Si with different texture periods ranging from 0.2 to 1 μm was calculated and averaged. The AM 1.5G spectrum was then weighted by (1-average R) as the input spectrum for Sentaurus to include the reflection or scattering effects.

## 2. Device Structure

**Figure 1:**
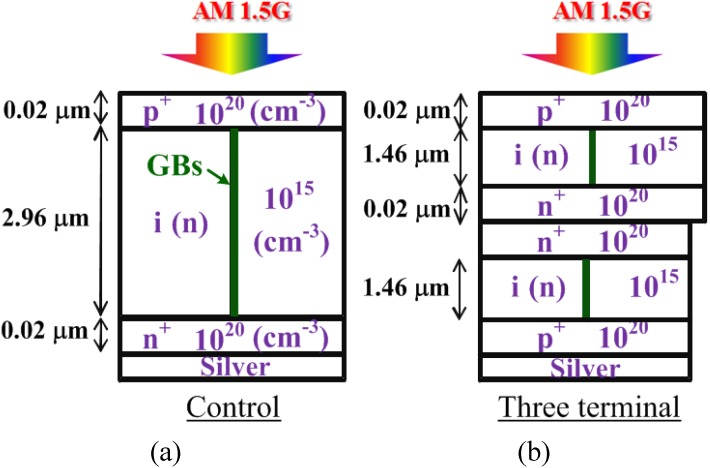
(**a**) The schematic structure of the typical two-terminal; and (**b**) the three-terminal μc-Si solar cells. The unintentionally doped i-layer is assumed to be n-type as observed in the experimental data.

In the numerical study, the 3-μm-thick p/i/n two terminal and p/i/n/n/i/p three-terminal μc-Si solar cells have been investigated. The doping concentrations and the device structures of the two-terminal (control) and the three-terminal μc-Si solar cells are shown in Figure 1. Light of AM1.5G solar spectrum is irradiated perpendicularly to the cell junction from the top side. Some material parameters used in this study were listed in Table 1. Both the control cells and the three-terminal cells have a thickness of 3 μm and all of the n-layers and p-layers were 0.02 μm. For both cells, the area of illumination is the same. Moreover, doping concentrations of n-layers, i-layers and p-layers are identical for both cells. The unintentionally doped i-layer is assumed to be n type with a concentration of 10^15^ cm^−3^. Since the practical μc-Si solar cells suffer from the recombination of photo-generated carriers at the grain boundaries [12], the grain boundaries (GBs) are set in the middle of the intrinsic layers as shown in Figure 1.

**Table 1:** Parameters used for microcrystalline silicon solar cells in this numerical study.

Input parameters	μc-Si	GB interface
Carriers mobility (cm^2^/Vs)	40(e)/10(h)	N/A
Peak of band-tail density of states	10^19^ (cm^−3^)	10^12^ (cm^−2^)
Peak of Gaussian density of states	10^14^ (cm^−3^)	10^10^ (cm^−2^)
Conduction band tail width (eV)	0.027	0.027
Valence band tail width (eV)	0.045	0.045

The reported μc-Si solar cells have a crystallinity (Xc) of about 60% [3]. The crystallinity of 60% from the Raman measurement corresponds to the volume fraction of ~90% [13]. Hence, the refractive index and extinction coefficient values of μc-Si in this study are calculated from 90% c-Si and 10% a-Si. Particulars of the grain boundary between the crystalline columns can be found in [14]. The TEM in [14] supports this idea. In [8], the assumption of the grain boundary region within a µc-Si layer achieves an accurate simulation for the experimental data. The conduction band/valence band tail states and two Gaussian dangling bond states have been set as the density of state in the grain boundary in [8]. Hence, we constructed our model of the control two-terminal cell based on such a concept, and the parameters for density of state are shown in Table 1. We can therefore obtain a simulation result close to the practical two-terminal cell. Furthermore, we could then show the influence of a three-terminal structure for the material of μc-Si.

The control cell is a typical pin solar cell. The three-terminal cell consists of two pin subcells connected in parallel, and each subcell is structurally the same as the control cell. The depletion region of the pin structure results in the built-in potential and helps the separation of photo-generated carriers [15]. For the control cell, the photo-generated holes and electrons would drift to the p-layer and n-layer, respectively. On the other hand, in the three-terminal cell, the photo-generated holes in the top subcell would drift to the top p-layer while photo-generated holes of the bottom subcell would drift to the bottom p-layer. Photo-generated electrons in the three-terminal cell would always drift to the n-layers between the subcells. Hence, unlike the tandem cell in serial configuration, the currents do not need to match for the three-terminal cell.

## 3. Results and Discussion

For the control cell, the p-layer is connected with the outer circuit by the top contact and acts as the anode, and the n-layer is connected with the outer circuit by the bottom contact and acts as the cathode. For the three-terminal cell, the two p-layers are joined and connected to the outer circuit, and act as the anode. The n-layer is connected with the outer circuit via the middle contact and acts as the cathode.

**Figure 2:**
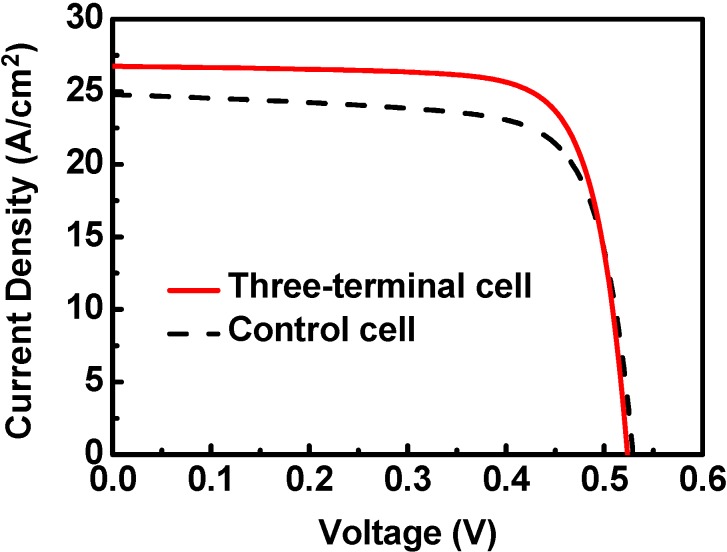
The J-V curve of the typical two-terminal (control) and the three-terminal μc-Si solar cells. The efficiency of the three-terminal cell is 10.8%, and the efficiency of the control cell is 9.7%.

The simulated J-V curves of the two-terminal cell and the three-terminal cell are shown in Figure 2. For the two-terminal control μc-Si solar cell, the efficiency was 9.7%, the V_OC_ was 0.530 V, and the J_SC_ was 24.8 mA/cm^2^. Meanwhile, the three-terminal cell could achieve a conversion efficiency of 10.8%, a V_OC_ of 0.523 V, and a J_SC_ of 26.8 mA/cm^2^. The three-terminal cell had a better efficiency as compared with the control cell.

For comparison, the performance of practical two-terminal cells demonstrated in [16] is described as following. A two-terminal μc-Si solar cell fabricated by HWCVD had an efficiency of 9.4%, a V_OC_ of 0.58 V, and a J_SC_ of 23.3 mA/cm^2^. On the other hand, the same efficiency of 9.4%, a V_OC_ of 0.53 V, and a J_SC_ of 25.1 mA/cm^2^ could be achieved when it was fabricated by PECVD. Our simulation results are close to these reported data.

**Figure 3:**
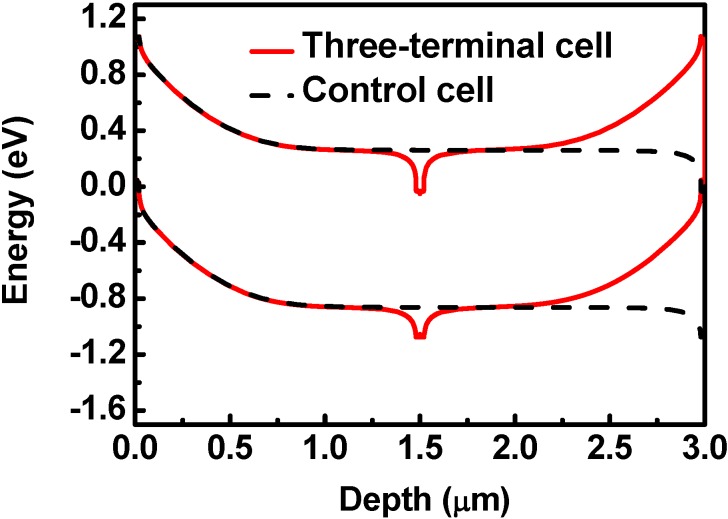
The band diagrams of the three-terminal cell and the control cell. The photo-generated electrons and holes drift to the n-layers and p-layers, respectively.

Figure 3 shows the 0-V band diagrams of the control cell and the three-terminal cell. There are two pin structures in the three-terminal cell. Meanwhile, there is only one pin structure in the control cell. The benefit of two pin structures in the three-terminal structure is described as follows.

**Figure 4:**
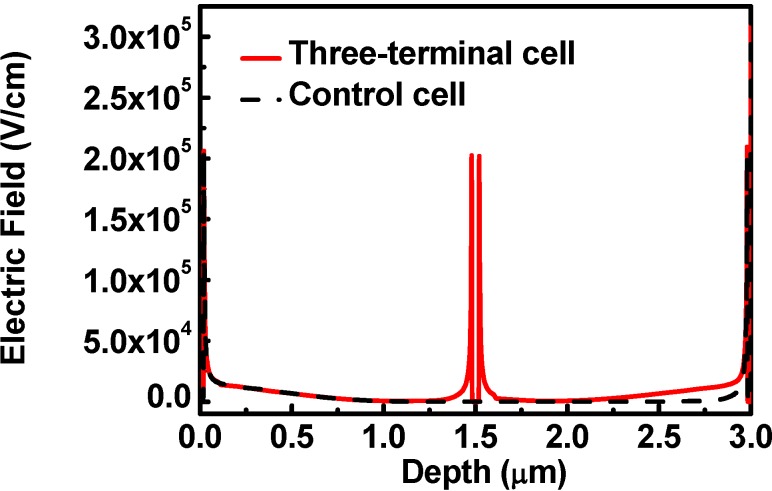
The magnitude of the electric field as a function of the depth. The electric field is widely boosted for the p/i junction.

The magnitude of the electric field as a function of the depth is shown in Figure 4. The electric field is widely boosted for the p/i junction, because the i-layer is assumed to be of an unintentionally doped n-type material. There are two p/i junctions for the three-terminal cell; meanwhile, there is only one p/i junction for the control cell. Hence, the electric field at the depths of both 0~1.0 μm and 2.0~3.0 μm is boosted for the three-terminal cell. However, the electric field is only boosted at a depth of 0~1.0 μm in the control cell. Compared to the control cell, the electric-field boosted region is wider in the three-terminal cell. In the electric-field boosted region, the photo-generated electron-hole pairs can be easily separated without recombination. In addition, there are two high-field i/n regions at the depths of 1.4~1.5 μm and 1.5~1.6 μm for the three-terminal cell, which is also superior to one high-field i/n region at the depth of 2.9~3.0 μm of the control cell. The enhancement of the efficiency of the three-terminal cell is achieved by reducing the recombination rate of the photo-generated carriers via a larger average electric field.

**Figure 5:**
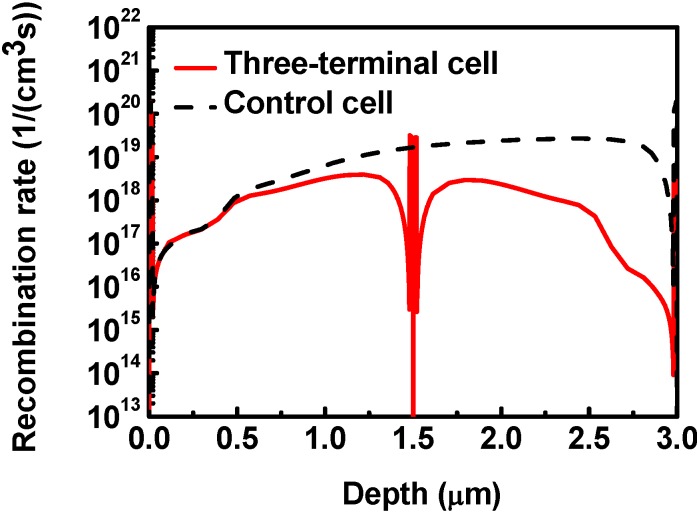
The recombination rate of the three-terminal cell and the two-terminal (control) cell. The three-terminal cell has a lower recombination rate than the control cell.

Figure 5 shows the recombination rate of the three-terminal cell and the control cell. As mentioned above, the larger average electric field in the three-terminal cell results in a smaller recombination rate. The three-terminal cell has a lower recombination rate than the control cell, and thus the efficiency of the three-terminal cell could be higher than the control cell.

In [17], when the three-terminal structure was applied to a-Si, both the J_SC_ and V_OC_ increased compared to the typical two-terminal cell. However, for μc-Si, the three-terminal structure only increased the J_SC_ but decreased the V_OC_. Since the subcells in the three-terminal cells are in parallel, the final J_SC_ is obtained with summation but the final V_OC_ would be between the V_OC_ of two subcells. It should be noted that V_OC_ would decrease as the thickness of the active layer increases especially for highly defective materials. Hence, for the a-Si, the top subcell of the three terminal cell with a half thickness as compared with the control two-terminal cell, could have an obviously higher V_OC_. The final V_OC_, which would be between the V_OC_ of the top subcell and the bottom subcell, of the three-terminal a-Si cell, could have larger V_OC_ than that of the two-terminal a-Si cell. On the other hand, the V_OC_ of μc-Si is less sensitive to the thickness due to higher quality in the bulk. The V_OC_ of the top subcell of the three-terminal μc-Si cell would not be obviously larger than that of the control. The bottom subcell has an even smaller V_OC_ due to insufficient absorption. Therefore, the final V_OC_ of the three-terminal μc-Si cell becomes smaller than the V_OC_ of the two-terminal μc-Si cell. The differing results between a-Si and μc-Si cases can provide insight into their material properties, and we may apply this information in order to design better structures in the future.

## 4. Conclusions

We included grain boundaries in the simulation of μc-Si solar cells. A new structure, the three-terminal μc-Si solar cell, was designed and simulated. The 3-μm-thick three-terminal μc-Si solar cell achieved an efficiency of 10.8%. The three-terminal structure appears to be promising for high efficiency photovoltaic applications by increasing the average electric field.
